# A case–control study in France showing that a pro-inflammatory diet is associated with a higher risk of breast cancer

**DOI:** 10.1038/s41598-021-95955-y

**Published:** 2021-08-23

**Authors:** Mariem Hajji-Louati, Emilie Cordina-Duverger, Nasser Laouali, Francesca-Romana Mancini, Pascal Guénel

**Affiliations:** Center for Research in Epidemiology and Population Health (CESP), Team Exposome and Heredity, U1018 Inserm, University Paris-Saclay, Institut Gustave Roussy, 94800 Villejuif Cedex, France

**Keywords:** Cancer, Cancer epidemiology, Breast cancer

## Abstract

Dietary regimens promoting inflammatory conditions have been implicated in breast cancer development, but studies on the association between pro-inflammatory diet and breast cancer risk have reported inconsistent results. We investigated the association between the inflammatory potential of diet and breast cancer risk in a case–control study in France including 872 breast cancer cases and 966 population controls. All women completed a food frequency questionnaire that was used to compute a Dietary Inflammatory Index (DII) based on the inflammatory weight of 33 dietary components. The DII ranged from a median of − 3.22 in the lowest quartile (anti-inflammatory) to + 2.96 in the highest quartile (pro-inflammatory). The odds ratio contrasting quartile 4 to quartile 1 was 1.31 (95% CI 1.00, 1.73; *p*-trend = 0.02). Slightly higher odds ratios were observed in post-menopausal women, particularly those with body mass index > 25 kg/m^2^ (odds ratio 1.62; 95% CI 0.92, 2.83; *p*-trend = 0.02), and among ever smokers (odds ratio 1.71; 95% CI 1.11, 2.65; p-trend 0.01). The analyses by breast cancer subtype showed that the DII was associated with breast tumors that expressed either the estrogen (ER) or progesterone (PR) hormone receptors or the Human Epidermal Growth Factor Receptor-2 (HER2), but no association was seen for the triple negative breast tumor subtype. Our results add further evidence that a pro-inflammatory diet is associated with breast cancer risk with possible effect variation according to tumor subtype.

## Introduction

Breast cancer is the most common cancer in women and the leading cause of cancer death among women, accounting for 2 million new cases and 0.6 million deaths worldwide in 2020^1^. While hormonal and lifestyle factors are known to contribute to the occurrence of breast cancer, considerable evidence suggests that chronic inflammation plays a crucial role in the pathogenesis of breast cancer and other cancers^2,3^. Diet is one of the main lifestyle-related factors that can modulate the inflammatory process^4–8^. Dietary components may have pro- or anti-inflammatory properties via modulation of inflammatory biomarker levels^9,10^. It has been shown that a high intake of fruits, vegetables and whole grain are associated with lower levels of inflammatory biomarkers^9^, while a high intake of red and processed meat resulted in an increased level of inflammatory biomarkers^11^.

In order to assess the inflammatory potential of the diet, Shivappa et al.^12^ have developed the Dietary Inflammatory Index (DII), a score based on the compilation of inflammatory weights of 45 food parameters combined with data of dietary intakes obtained from food frequency questionnaires. In the Shivappa approach^12^, the dietary intake is standardized on the mean and standard deviation of dietary intakes of 45 food parameters available in a composite dietary database from eleven countries to facilitate international comparisons. Using a slightly different approach, an adapted DII was developed by van Woudenbergh et al.^13^ where the intake of dietary components is standardized on the means and standard deviations of dietary intakes in the population under study without reference to an external database. Recently, other authors used this adapted DII^14–16^ in combination with the inflammatory weights of dietary components proposed by Shivappa^12^.

The DII has been used in epidemiological studies on breast cancer with mixed and inconsistent results. While several studies reported a positive association between a pro-inflammatory diet and breast cancer^17–24^, others found no such association^25–31^. It is also unclear whether a pro-inflammatory diet may affect breast cancer risk differently according to menopausal status, body weight or other risk factors that may modify the association between DII and breast cancer. Moreover, it is possible that the association of inflammatory diet with breast cancer differs by tumor subtype defined by expression of ER, PR and HER2 receptors in tumor tissue, but inconsistent findings have been reported in a limited number of studies^20,26,29^.

In the present study, we investigated the role of dietary inflammation in relation to breast cancer risk among women in two regions of France, using the adapted DII score. We will also examine whether the association between DII and breast cancer is modified according to selected breast cancer risk factors and by breast tumor subtype.

## Material and methods

### Study population

We conducted a population-based case–control study (CECILE study) in Ille-et-Vilaine and Côte d’Or, two administrative areas (*départements*) in the western and eastern part of France, respectively.

Cases were patients aged 25 to 75 years old with incident invasive or in situ breast cancer diagnosed between April 2005 and March 2007 in the two study areas. Cases were actively searched in the main cancer treatment hospital of each *département*, and from other smaller public or private hospitals treating breast cancer patients in these areas in an effort to recruit all incident cases of breast cancer during the study period. Among the 1553 eligible cases selected during the study period, 1232 cases (79.3%) were recruited for the study and were contacted for an in-person interview.

Controls were women frequency-matched to the cases by 10-year age group selected from the general population of each study area. To form the control group, women living in a random sample of private homes were contacted by phone and invited to participate in the study if they had no previous history of breast cancer. To account for possible differential participation rate across categories of socio-economic status (SES), quotas by SES were used to obtain a distribution by SES in the control group similar to that of the female general population, but socioeconomic status was not used as a criterion for matching cases and controls in our study. Among the 1731 controls identified by phone fulfilling eligibility criteria for SES, 1317 (76%) agreed to participate.

### Data collection

An in-person interview of the cases and the selected controls was conducted with trained interviewers by means of a standardized questionnaire. We collected information on socio-demographic characteristics, family history of cancer, personal medical history, reproduction, lifestyle-related factors, as well as occupational and residential history. At the end of the interview, women were asked to complete a food frequency questionnaire (FFQ) and to return it to the research team in a prepaid envelope.

### Assessment of dietary intake

In the FFQ we sought to obtain information on the dietary intake of 153 food items in the year prior to diagnosis for the cases or prior to recruitment for the controls. Women were asked to report the frequency of intake of each food item in times per day, week or month, as well as the portion size (small, medium or large) using food photographs. Total energy and nutrient intakes were estimated using a food composition table specifically developed for the study based on the CIQUAL-2013 French food composition database^32^ and on the NutriNet-Santé composition table^33^. The polyphenol intake was estimated using the Phenol-Explorer database^34^ and the EPIC Nutrient Database^35^. From the 153 food items available from the FFQ, we used 33 dietary components with inflammatory weight available in Shivappa^12^ to calculate the DII (see Table 2).

In the present analysis, we excluded women who did not complete the dietary questionnaire (341 cases; 332 controls). In addition, women with a ratio of dietary energy intake to energy requirement below the 1st or above the 99th percentile (19 cases; 19 controls) were also excluded. In total, the present analysis included 872 cases (70.8%) and 966 controls (73.3%).

The study was approved by a French Ethics Committee (CCPPRB Kremlin-Bicêtre January 2015, Nr 04–53), and was conducted in accordance with the French regulations for medical research. A signed informed consent was obtained from all study subjects.

### The dietary inflammatory index (DII)

In order to obtain the DII score for each subject, we used the approach described by Laouali et al.^14^ that combines the calculation of the adapted DII proposed by van Woudenbergh et al.^13^ and the inflammatory weight of the dietary components proposed by Shivappa et al.^12^. First, the dietary intake of each 33 dietary components was adjusted for total energy intake using the residual method^36^; second, the energy-adjusted intake was standardized by subtracting the mean and dividing by the standard deviation of the dietary intake calculated over the whole study sample; third, the standardized energy-adjusted intake of the dietary component was multiplied by its corresponding inflammatory weight; fourth, the weighted standardized energy-adjusted intakes of the 33 dietary components were summed to obtain the overall DII for each subject. A high DII indicates a pro-inflammatory diet and a low DII indicates an anti-inflammatory diet.

### Statistical analysis

The DII score was analyzed as a categorical variable in quartiles based on the distribution among controls. Odds ratio (ORs) and 95% confidence intervals (CIs) were calculated using unconditional logistic regression models adjusting for age (continuous variable), department (Ille-et-Vilaine; Côte d’Or), history of benign breast disease (yes/no), family history of breast cancer in first-degree relatives (yes/no), age at menarche (≤ 11, 12, 13, 14, ≥ 15 years), parity (0, 1, 2, 3, ≥ 4), age at first full-term pregnancy (< 22, 22–24, 25–27, > 27 years), duration of breast feeding (0, < 26, 26–52, ≥ 52 weeks), menopausal status (pre-menopausal and post-menopausal), menopausal hormone therapy (yes/no), oral contraceptive use (never/ever), and body mass index (BMI)(< 25 , ≥ 25 kg/m^2^). For all covariates missing values were < 1.5% and therefore were imputed by the corresponding mode value. Additional adjustments for socioeconomic status and education were also made, but these had only marginal influence on the odds ratios (results not shown).

The *p*-values for trend were determined by fitting models using the median values of each quartile as a quantitative variable in the model.

We studied the association between the DII and breast cancer in pre- and post-menopausal women separately. In addition, we conducted analyses stratified by area of residence (Côte d’Or, Ille-et-Vilaine), BMI, physical activity, smoking status and alcohol consumption. Further analysis was performed by breast cancer subtype based on estrogen-receptor (ER), progesterone receptor (PR) and Human Epidermal Growth Factor Receptor-2 (HER2). Breast cancer tumors were classified as hormone-receptor positive (i.e., ER-positive or PR-positive and HER2-negative, equivalent to the Luminal A molecular subtype), HER2-positive (HER2-positive regardless of ER and PR status, equivalent to the Luminal B and HER2-enriched molecular subtypes), and triple negative tumors (ER-negative, PR-negative and HER2-negative). All analyses were carried out using SAS (version 9.4; SAS Institute Inc., Cary, NC, USA).

### Ethics approval

The protocol of the CECILE study was approved by the Ethic Committee Kremlin-Bicêtre (CCPPRB Jan 2005) and by the National Data Protection Commission (CNIL Dec 2004).

### Consent to participate

All participants signed informed consent before inclusion.

## Results

The distribution of cases and controls by age, study area and breast cancer risk factors are shown in Table 1. As expected, breast cancer risk was associated with a family history of breast cancer, personal history of benign breast disease, early age at menarche, low parity, late age at first full-term pregnancy, current use of menopausal hormone therapy, and low BMI in pre-menopausal women. In our data, no statistically significant association was detected with use of oral contraceptive, breastfeeding, smoking status, alcohol consumption, physical activity and BMI in post-menopausal women.

**Table 1:** Characteristics of the study population and odds ratios associated with well-established breast cancer risk factors. The CECILE study, France, 2005–2007.

	Cases (N = 872) n (%)	Controls (N = 966) n (%)	OR^a^	95% CI
Study area*				
Côte d'Or	239 (27.4)	307 (31.8)	_	_
Ille-et-Vilaine	633 (72.6)	659 (68.2)	_	_
Age*				
25–34	32 (3.67)	33 (3.4)	_	_
35–44	133 (15.2)	129 (13.5)	_	_
45–54	263 (30.2)	310 (32.1)	_	_
55–64	267 (30.6)	268 (27.7)	_	_
65 +	177 (20.3)	226 (23.4)	_	_
**Family history of breast cancer**				
No	715 (82.0)	870 (90.1)	1.00	Reference
Yes	157 (18.0)	96 (9.9)	1.92	1.45, 2.55
**History of benign breast disease**				
No	508 (58.3)	668 (69.1)	1.00	Reference
Yes	364 (41.7)	298 (30.9)	1.51	1.24, 1.85
**Age at menarche (years)**				
≤ 11	167 (19.2)	142 (14.7)	1.00	Reference
12	222 (25.5)	237 (24.5)	0.79	0.59, 1.07
13	203 (23.3)	214 (22.2)	0.81	0.59, 1.10
14	156 (17.9)	203 (21.0)	0.68	0.49, 0.93
≥ 15	124 (14.2)	170 (17.6)	0.61	0.44, 0.86
**Oral contraceptive use**				
Never	614 (70.4)	691 (71.5)	1.00	Reference
Ever	258 (29.6)	275 (28.5)	1.00	0.78, 1.28
**Parity**				
Nulliparous	105 (12.0)	58 (6.0)	1.00	Reference
1 FTP	140 (16.1)	117 (12.1)	0.63	0.41, 0.99
2 FTP	351 (40.3)	372 (38.5)	0.56	0.38, 0.83
3 FTP	200 (22.9)	289 (29.9)	0.43	0.29, 0.63
≥ 4 FTP	76 (8.7)	130 (13.5)	0.38	0.24, 0.59
**Age at FFTP (years) among parous women**				
< 22	169 (19.4)	245 (25.4)	1.00	Reference
22–24	222 (25.5)	286 (29.6)	1.05	0.80, 1.39
25–27	187 (21.4)	230 (23.8)	1.11	0.83, 1.49
> 27	189 (21.7)	147 (15.2)	1.63	1.18, 2.24
**Breastfeeding (weeks) among parous women**				
0	369 (42.3)	431 (44.6)	1.00	Reference
< 26	293 (33.6)	339 (35.1)	1.01	0.81, 1.26
26–52	69 (7.9)	91 (9.4)	0.93	0.64, 1.33
≥ 52	36 (4.1)	47 (4.9)	1.05	0.65, 1.71
**Current use of MHT (among postmenopausal women)**				
No	415 (47.6)	495 (51.2)	1.00	Reference
Yes	111 (12.7)	96 (9.9)	1.44	1.04, 1.99
**Menopausal status**				
Pre-menopausal women	346 (39.7)	375 (38.8)	1.00	Reference
Post-menopausal women	526 (60.3)	591 (61.2)	0.96	0.70, 1.32
**Smoking status**				
Never	524 (60.1)	589 (61.0)	1.00	Reference
Former	200 (22.9)	235 (24.3)	0.92	0.72, 1.16
Current	148 (17.0)	142 (14.7)	1.10	0.83, 1.47
**Alcohol consumption (glasses per week)**				
0–3	669 (76.7)	722 (74.7)	1.00	Reference
4–7	117 (13.4)	149 (15.4)	0.81	0.61, 1.07
8–14	52 (6.0)	62 (6.4)	0.92	0.62, 1.38
≥ 14	34 (3.9)	33 (3.4)	1.07	0.64, 1.79
**Physical activity**				
At least 1 h/week for 1 year	617 (70.8)	710 (73.5)	1.00	Reference
No	255 (29.2)	256 (26.5)	0.83	0.66, 1.03
**BMI (kg/m**^**2**^**) among pre-menopausal women**				
< 18.5	22 (6.4)	8 (2.1)	2.38	0.99, 5.75
18.5–25	253 (73.1)	249 (66.4)	1.00	Reference
25–30	50 (14.5)	81 (21.6)	0.57	0.37, 0.87
≥ 30	21 (6.1)	37 (9.9)	0.61	0.33, 1.11
**BMI (kg/m**^**2**^**) among post-menopausal women**				
< 18.5	12 (2.3)	10 (1.7)	1.26	0.52, 3.07
18.5–25	274 (52.1)	311 (52.6)	1.00	Reference
25–30	159 (30.2)	173 (29.3)	1.16	0.87, 1.54
≥ 30	81 (15.4)	97 (16.4)	1.06	0.74, 1.52

*Matching variables.

*FTP* full term pregnancy, *FFTP* first full-term pregnancy, *MHT* menopausal hormonal treatment, *BMI* body mass index, *OR* odds ratio, *CI* confidence interval.

^a^Odds ratios adjusted for all variables in the table.

The median DII and the median intake of the dietary components in each DII quartile are shown in Table 2. The median DII increased from − 3.22 in quartile 1 (most anti-inflammatory) to + 2.96 in quartile 4 (most pro-inflammatory), corresponding to a variation of about 200%. Consistently, intakes of anti-inflammatory components such as tea, onions, flavonoids, vitamin C, fibers, and ß-carotene decreased sharply from quartile 1 to quartile 4, while intakes of pro-inflammatory components such as cholesterol and saturated fat increased.

**Table 2:** Median nutritional intake per day by quartile of DII among controls.

	Q1	Q2	Q3	Q4	%Variation (Q4-Q1)/|Q1| (%)
	N = 241	N = 242	N = 241	N = 242	
**DII (median)**	**− 3.22**	**− 0.6**	**1.05**	**2.96**	** + 192**
**Dietary components**					
Tea (ml)	76	23	32	7	− 91
Onion (g)	43	14	14	7	− 84
Flavonols (mg)	25	14	10	7	− 72
Flavanones (mg)	9	5	4	3	− 67
Isoflavones (mg)	0.09	0.04	0.03	0.03	− 67
β-Carotene (µg)	7475	5282	3689	3292	− 56
Vitamin C (mg)	226	154	126	102	− 55
Fibre (g)	34	23	19	17	− 50
n-3 Fattyacids (g)	2	1	1	1	− 50
Thiamin (mg)	2	1	1	1	− 50
Vitamin B12 (µg)	9	7	4	5	− 44
Folate (µg)	507	354	281	269	− 47
Vitamin E (mg)	21	17	14	13	− 38
Iron (mg)	11	8	7	7	− 36
Magnesium (mg)	345	256	216	229	− 34
Alcohol (g)	3	3	3	2	− 33
Zinc (mg)	14	11	9	10	− 29
Anthocyanidins (mg)	23	19	15	17	− 26
Selenium (µg)	69	59	48	54	− 22
Flavan-3-ol (mg)	34	26	26	27	− 21
Niacin (mg)	17	15	13	14	− 18
Carbohydrate (g)	171	138	128	143	− 16
n-6 Fattyacids (g)	9	8	7	8	− 11
Protein (g)	75	65	58	67	− 11
Caffeine (mg)	138	134	132	131	− 5
Vitamin B6 (mg)	2	2	1	2	0
Riboflavin (mg)	2	2	1	2	0
Flavones (mg)	2	1	1	2	0
Vitamin D (µg)	3	3	2	3	0
Vitamin A (µg)	364	321	284	386	6
MUFA (g)	30	26	26	32	7
Cholesterol (mg)	228	214	201	264	16
Saturated fat (g)	27	26	27	39	44

The CECILE study, France, 2005–2007.

*MUFA* monounsaturated fatty acids, *PUFA* polyunsaturated fatty acids.

Table 3 shows the odds ratios for breast cancer by DII quartile. Among all women, the odds ratio was 1.05 (95% CI 0.80, 1.39) in quartile 2, and increased to 1.39 (95% CI 1.06, 1.83) and 1.31 (95% CI 1.00, 1.73) in quartiles 3 and 4, respectively (p-trend = 0.02). In pre-menopausal women, the odds ratios increased by about 20% in quartiles 3 and 4, although none reached statistical significance. In post-menopausal women, odds ratios reached slightly higher values in quartiles 3 and 4, with a significant p-trend (p = 0.02). No significant interaction between DII and menopausal status was seen (p-interaction = 0.49).

**Table 3:** Odds ratios (ORs) and 95% confidence intervals (CIs) for breast cancer associated with Dietary Inflammatory Index (DII).

	Case/control	Quartiles of DII								
		Q1		Q2		Q3		Q4		*p*-trend
		< − 1.67		≥ − 1.67 < 0.20		≥ 0.20 < 1.86		≥ 1.86		
		OR	95% CI	OR	95% CI	OR	95% CI	OR	95% CI	
All women	872/966	1.00	Reference	1.05	0.80, 1.39	1.39	1.06, 1.83	1.31	1.00, 1.73	0.02
Pre-menopausal women*	346/375	1.00	Reference	1.02	0.63, 1.65	1.21	0.76, 1.92	1.24	0.80, 1.93	0.27
Post-menopausal women	526/591	1.00	Reference	1.08	0.76, 1.54	1.51	1.07, 2.13	1.38	0.96, 1.99	0.02

All women and stratification by menopausal status. The CECILE study, France, 2005–2007.

*P* trend determined from the model using the median value in each quartile as a continuous variable.

Odds ratios adjusted for study area, age as continuous variable, history of benign breast, family history of breast cancer in first-degree relatives, age at menarche, parity, age at first full-term pregnancy, breast feeding, menopausal status, current use of hormone therapy, oral contraceptive use and body mass index.

*Not adjusted for current use of hormone therapy.

*P*-value for interaction (DII x menopausal status): 0.49.

Table 4 shows odds ratios by study area and by strata of selected breast cancer risk factors. Among pre-menopausal women, no increasing trend of the odds ratios with increasing DII was observed in either BMI strata (≤ 25, > 25 kg/m^2^), while among post-menopausal the odds ratios increased with DII in overweighed women (p-trend = 0.02) but not in leaner women (p-trend = 0.46). The stratification according to smoking status showed a significant trend among ever smokers (p-trend = 0.01), but not among never smokers (p-trend = 0.45). However, none of the interaction tests between DII and breast cancer risk factors shown in Table 4 was significant. No noticeable difference of the odds ratios for DII was observed between strata of physical activity and alcohol drinking.

**Table 4:** Odds Ratios (ORs) and 95% Confidence Intervals (CIs) for breast cancer associated with the Dietary Inflammatory Index (DII).

	Case/control	Quartiles of DII								*p-*trend	*P-*interaction
		Q1		Q2		Q3		Q4			
		OR	95% CI	OR	95% CI	OR	95% CI	OR	95% CI		
**Department**											
Côte d'Or	239/ 307	1.00	Reference	1.56	0.89, 2.72	1.82	1.06, 3.12	1.33	0.77, 2.31	0.24	
Ille-et-Vilaine	633/ 659	1.00	Reference	0.89	0.64, 1.24	1.24	0.90, 1.71	1.29	0.93, 1.80	0.05	0.95
**BMI (pre-menopausal)**											
≤ 25 kg/m^2^	275/257	1.00	Reference	1.19	0.68, 2.10	1.43	0.83, 2.47	1.36	0.81, 2.29	0.21	
> 25 kg/m^2^	71/118	1.00	Reference	0.60	0.22, 1.69	0.68	0.26, 1.75	0.98	0.40, 2.36	0.97	0.61
**BMI (post-menopausal)**											
≤ 25 kg/m^2^	286/321	1.00	Reference	0.72	0.44, 1.18	1.10	0.69, 1.76	1.10	0.67, 1.79	0.46	
> 25 kg/m^2^	240/270	1.00	Reference	1.61	0.96, 2.70	2.23	1.32, 3.77	1.62	0.92, 2.83	0.02	0.35
**Physical activity**											
Inactive	255/ 256	1.00	Reference	1.26	0.69, 2.30	2.17	1.25, 3.79	1.43	0.83, 2.46	0.09	
Active	617/ 710	1.00	Reference	0.97	0.70, 1.34	1.13	0.82, 1.55	1.28	0.92, 1.77	0.12	0.49
**Tobacco smoking**											
Never smokers	524/ 589	1.00	Reference	0.91	0.63, 1.32	1.28	0.90, 1.81	1.04	0.72, 1.50	0.45	
Ever smokers	348/ 377	1.00	Reference	1.27	0.81, 1.97	1.44	0.91, 2.26	1.71	1.11, 2.65	0.01	0.43
**Alcohol drinking**											
≤ 1 glass/week	345/ 351	1.00	Reference	0.88	0.55, 1.42	1.52	0.97, 2.38	1.38	0.89, 2.13	0.05	
> 1 glass/week	527/ 615	1.00	Reference	1.17	0.82, 1.67	1.36	0.96, 1.92	1.23	0.86, 1.76	0.16	0.93

Stratification by selected breast cancer risk factors. The CECILE study, France, 2005–2007.

*P* trend determined from the model using the median value in each quartile as a quantitative variable.

Odds ratios adjusted for study area, age at reference date, history of benign breast, family history of breast cancer in first-degree relatives, age at menarche, parity, age at first full-term pregnancy, breast feeding, menopausal status, current use of hormone therapy, oral contraceptive use and body mass index.

No adjustment on the stratification variable.

The odds ratios for DII associated with breast cancer subtypes are shown in Table 5. The odds ratios for hormone receptor-positive tumors (ER-positive or PR-positive) increased with increasing DII (p-trend = 0.03). We also found that the odds ratios in DII quartiles 2 to 4 increased in HER2-positive tumors (p-trend = 0.03). No trend between DII and triple negative breast tumors was observed.

**Table 5:** Odds Ratios (ORs) and 95% Confidence Intervals (CIs) for the association between Dietary Inflammatory Index (DII) and breast cancer by Hormone Receptors Status.

Hormone receptor status	N	Quartiles of DII								
		Q1		Q2		Q3		Q4		*p*-trend
		< − 1.67		≥ —1.67 < 0.20		≥ 0.20 < 1.86		≥ 1.86		
		OR	95% CI	OR	95% CI	OR	95% CI	OR	95% CI	
HR-positive*	687	1.00	Reference	1.02	0.75, 1.38	1.27	0.95, 1.70	1.34	1.00, 1.80	0.03
HER2-positive‡	94	1.00	Reference	1.82	0.88, 3.76	2.20	1.08, 4.49	2.18	1.07, 4.41	0.03
TN†	73	1.00	Reference	1.17	0.56, 2.43	1.21	0.59, 2.47	0.94	0.45, 1.96	0.92

The CECILE study, France, 2005–2007.

*HR-positive: Hormone receptor positive tumors, i.e. (ER-positive or PR-positive) and HER2-negative.

^‡^HER2-positive regardless of the ER or PR status.

^†^TN triple negative (ER-negative and PR-negative and HER2-negative).

*p-*trend determined from the model using the median value in each quartile as a continuous variable.

Odds ratios adjusted for study area, age at reference date, history of benign breast, family history of breast cancer in first-degree relatives, age at menarche, parity, age at first full-term pregnancy, breast feeding, menopausal status, current use of hormone therapy, oral contraceptive use and body mass index.

## Discussion

Our results suggest that a pro-inflammatory diet is associated with an increased risk of breast cancer in French women. This association appeared to be slightly stronger in post-menopausal than in pre-menopausal women, particularly those who were overweighed. It was also slightly stronger in ever smokers as compared to never smokers. Our results also show that a pro-inflammatory diet increases the risk of the most frequent breast tumor subtypes, i.e. tumors that are positive for either ER, PR or HER2-positive tumors, but no association was found for the triple negative (ER-negative, PR-negative and HER2-negative) breast cancers.

### Literature review

There is strong evidence that chronic inflammation plays a major role in breast carcinogenesis^2,37,38^, and several prospective studies have shown that circulating C-reactive protein (CRP), a systemic inflammation biomarker, is associated with an increased risk of breast cancer^39^. Besides, the inflammatory potential of the diet has been demonstrated by studies showing that a high intake of refined carbohydrates, saturated fatty acids and low intake of dietary fiber are associated with increased levels of circulating pro-inflammatory markers, while nutrients like β-carotene, magnesium and flavonoids may inhibit and suppress the activation of certain pro-inflammatory markers^7,9,10^. Based on these results, several prospective cohorts^17,19,25–27^ or case–control studies^18,20–24,28,29^ have sought to demonstrate that a pro-inflammatory diet is associated with breast cancer risk, using the dietary inflammatory index developed by Shivappa et al.^12^. Case–control studies generally reported that a pro-inflammatory diet was associated with an increased risk of breast cancer,with one exception^29^, but considerable heterogeneity between studies was observed^21,40,41^. Conversely, cohort studies reported no^25–27^ or only marginally increased risks^17^. Further scrutiny of dietary patterns and of covariate distributions between study populations would be useful to understand these discrepancies.

### Effect modification according to covariates

Although the association of the DII with breast cancer was slightly higher in post- than in pre-menopausal women in our data, the differences were very small. Among studies that examined women separately by menopausal status, some reported stronger associations in pre-menopausal women^21,22,40^, others in post-menopausal women^19,23^, and others reported no clear difference between groups^18^.

Among post-menopausal women, we found that the association between DII and breast cancer was more pronounced in overweighed than in leaner women. Previous studies also reported slightly stronger associations of DII with breast cancer in overweighed than in leaner women^17,18,22,24,30^, yet this was not evaluated according to menopausal status. Obesity is associated with chronic inflammation due to the release of pro-inflammatory mediators by adipose tissue^42,43^. It has also been shown that the release of pro-inflammatory markers by the adipose tissue is stimulated by excessive intake of macronutrients^44^. It is thus possible that the pro-inflammatory condition conferred by overweight combines with that of a pro-inflammatory diet to enhance the risk of cancer. The estrogen production in adipose tissue of menopausal women is another possible explanation for a synergistic effect of obesity and pro-inflammatory diet on breast cancer risk^42,45^.

We found that the DII was more strongly associated with breast cancer in ever than in never smokers. Although no effect modification by smoking status was observed in the Spanish case–control study on DII and breast cancer^28^, a similar effect was reported in studies examining the association between DII and head and neck^46^ or thyroid cancer^47^. Complex immune modulatory effects occur among smokers including induction of a chronic inflammatory process^48^. When concurrent with other inflammatory conditions, high inflammatory potential of the diet might therefore increase the body inflammatory load and the risk of developing breast cancer. The combined effects of smoking and dietary inflammation on breast cancer risk should may be worthy of further investigation.

### Breast cancer subtypes

We found that a pro-inflammatory diet was associated with tumors subtypes that express the hormone receptors ER or PR (*p-trend* 0.03), or those that are positive for HER2 (*p-trend* 0.03), corresponding to the molecular subtypes Luminal A, Luminal B and HER2-enriched tumors. Conversely, we found no evidence of an association between DII and Triple Negative breast tumors defined by the absence of ER, PR and HER2 receptors, although the relatively small number of cases with this subtype may explain this negative finding. Triple negative tumors stand out for their aggressive behavior, and it is possible that they do not share the same etiological pathways as other breast cancer subtypes^49^.

To our knowledge, results for breast cancer subtypes based on the ER, PR and HER2 receptor status have been reported by only two studies^26,28^. Our findings are in line with those of Obón-Santacana et al.^28^, who reported an odds ratios of 1.22 (95% CI 0.95, 1.55) for ER-positive/PR-positive tumors, 1.56 (95% CI 1.01, 2.04) for HER2-positive tumors, and 0.99 (95% CI 0.56, 1.75) for triple negative tumors. In the Women’s Health Initiative cohort study, Tabung reported null results for all tumor subtypes^26^. Other studies which evaluated the association of DII with breast cancer subtypes based only on the ER/PR receptor status found no clear difference between tumor subtypes^20,22,23,29^. Investigating the links between the inflammatory potential of the diet and tumorigenesis driven by hormonal or HER2 pathway might be helpful to see whether breast cancer subtypes are affected differently by pro-inflammatory diet.

### Strengths and limitations

Previous studies examining the role of dietary inflammation in breast cancer have used the DII score of Shivappa et al.^12^ to classify dietary habits into pro- or anti-inflammatory. In our study, we computed an adapted DII following an approach that was used previously in studies on gastric cancer^50^, diabetes^14^, or hypertension^15^. This procedure slightly differs from that of Shivappa. First, we did not use the inflammatory weight for total fat to compute the DII because the 3 components of dietary fat (saturated, monounsaturated, and polyunsaturated fats) were also included separately. Second, the weight used for alcohol was different because alcohol was considered to be anti-inflammatory for intake below 40 g/day, and was assumed to have no inflammatory effect for intake above 40 g/day^51,52^. Third, the intake of food components in our study subjects was standardized by using the mean and standard deviation of each component in our study population, while the DII of Shivappa was standardized using a regional world-wide database taken as a referent population. The adapted DII was chosen in our study because this approach allowed us to perform the analyses without having to rely on a worldwide food consumption database, and because the objective was not to compare the inflammatory potential of the diet across populations, but to assess whether the inflammatory potential of the diet was associated with breast cancer risk in our population of French women. In addition, in a study on type 2 diabetes among French women comparing different approaches, similar results were observed regardless of the DII^14^.

Our study has some limitations. First, selection and recall bias cannot be excluded as in any study using a case–control study design. In our study, selection bias was minimized as we aimed to include all incident cases that occurred during the study period among women living in two well-defined geographical areas, and by including controls who were representative of the population from which the cases arose, using quotas by socioeconomic status to avoid differential participation rates. Possible recall bias was also reduced by the use of standardized questionnaires. Exposure misclassification on dietary intake was also controlled, at least in part, by excluding subjects with implausible dietary recall. To calculate the DII, we relied on the 33 food parameters that were available out of the 45 pre-defined parameters. However, it was shown that the predictive ability of DII score does not change with the decrease of the number of dietary parameters^26,53^. Finally, unmeasured dietary factors that are potentially associated with the DII may have led to residual confounding.

In conclusion, our findings add to the evidence that a pro-inflammatory diet increases the risk of breast cancer, especially among overweight post-menopausal women. Our data also suggest that a pro-inflammatory diet affects the most frequent breast cancer subtypes, with the exception of triple negative breast cancer that may not share the same etiological pathways. Dietary anti-inflammatory regimens are beneficial for disease prevention at large and might be considered as a means for preventing breast cancer.

## Abbreviations

DII: Dietary inflammatory index
BMI: Body mass index
ER: Estrogen receptor
PR: Progesterone receptor
HER2: Human epidermal growth factor receptor 2
